# Engineering the fragment crystallizable (Fc) region of human IgG1 multimers and monomers to fine-tune interactions with sialic acid-dependent receptors

**DOI:** 10.1074/jbc.M117.795047

**Published:** 2017-06-15

**Authors:** Patricia A. Blundell, Ngoc Phuong Lan Le, Joel Allen, Yasunori Watanabe, Richard J. Pleass

**Affiliations:** From the ‡ Department of Parasitology, Liverpool School of Tropical Medicine, Liverpool, L3 5QA, United Kingdom and; the § Department of Biochemistry, University of Oxford, Oxford OX1 3QU, United Kingdom

**Keywords:** Fc-gamma receptor, glycosylation, immunoglobulin G (IgG), N-linked glycosylation, sialic acid, Fc, intravenous immunoglobulin, sialoadhesin, siglec-1

## Abstract

Multimeric fragment crystallizable (Fc) regions and Fc-fusion proteins are actively being explored as biomimetic replacements for IVIG therapy, which is deployed to manage many diseases and conditions but is expensive and not always efficient. The Fc region of human IgG1 (IgG1-Fc) can be engineered into multimeric structures (hexa-Fcs) that bind their cognate receptors with high avidity. The critical influence of the unique *N*-linked glycan attached at Asn-297 on the structure and function of IgG1-Fc is well documented; however, whether the *N*-linked glycan has a similarly critical role in multimeric, avidly binding Fcs, is unknown. Hexa-Fc contains two *N*-linked sites at Asn-77 (equivalent to Asn-297 in the Fc of IgG1) and Asn-236 (equivalent to Asn-563 in the tail piece of IgM). We report here that glycosylation at Asn-297 is critical for interactions with Fc receptors and complement and that glycosylation at Asn-563 is essential for controlling multimerization. We also found that introduction of an additional fully occupied *N*-linked glycosylation site at the N terminus at position 1 (equivalent to Asp-221 in the Fc of IgG1) dramatically enhances overall sialic acid content of the Fc multimers. Furthermore, replacement of Cys-575 in the IgM tail piece of multimers resulted in monomers with enhanced sialic acid content and differential receptor-binding profiles. Thus insertion of additional *N*-linked glycans into either the hinge or tail piece of monomers or multimers leads to molecules with enhanced sialylation that may be suitable for managing inflammation or blocking pathogen invasion.

## Introduction

Multimeric Fc^2^ and Fc-fusion proteins are increasingly being explored for novel drug, diagnostic, and vaccine approaches (1–3). One potential area is their development as biomimetic replacements for intravenous immunoglobulin (IVIG) therapy. IVIG is a successful biological with Food and Drug Administration approval for treating idiopathic thrombocytopenic purpura (ITP), Kawasaki disease, Guillain–Barré syndrome, Graves ophthalmopathy and numerous polyneuropathies (4, 5). IVIG is increasingly viewed by clinicians as a last resort “cure-all” for a plethora of other diseases including anemias, arthritides, lupus, transplant rejection, abortion, and chronic pain, especially when these are non-responsive to conventional therapies (4, 5).

The global shortage and demand for IVIG is compounded by a number of other inadequacies with the current drug, the most significant being its dependence on human donors for its production, raising safety issues and greatly adding to cost. To add insult to injury, it is believed that less than 5% of the injected product is therapeutically active, leading to a requirement for high dosage (2g/kg) (6, 7). Consequently, IVIG is expensive, and adverse events caused by excessive IVIG loading are not uncommon (4, 5). Hence there is an urgent clinical need to develop synthetic replacements for IVIG for use in the clinic.

The mechanism of action of IVIG is incompletely understood. Although both Fab′_2_ and Fc-mediated mechanisms may be involved, in humans the infusion of Fc fragments is sufficient to ameliorate ITP (8). These Fc fragments inhibit harmful inflammation by engaging classical and non-classical Fc receptors and/or by forming complexes *in vivo* that allow IVIG to interact with such receptors with greater avidity, thus mediating more potent anti-inflammatory effects (7, 9–11). The exact receptors or combinations of receptors involved are not definitively known, although both classical (type 1) (*e.g.* FcγRIIB and FcγRIIIA) and non-classical (type 2) FcγRs (*e.g.* DC-SIGN, CD22, and FcRL5) have been implicated in its therapeutic efficacy (1, 12, 13).

Based on our earlier findings and those of other groups that Fc multimers can also induce tolerance (7, 10, 13, 15, 16), a number of different approaches to Fc multimerization are being actively investigated (1–3, 17). One approach utilizing the hinge region of human IgG2 generates laddered sequential multimers of diverse molecular masses when introduced into a mouse IgG2a-Fc backbone (18). These higher-order multimers, termed “stradomers” bind strongly to low-affinity FcγRs and SIGN-R1 and were shown to protect animals from collagen-induced arthritis, ITP, inflammatory neuropathy, and autoimmune myasthenia gravis (18–20).

We took an alternative approach to multimerization by fusing the 18-amino acid tail piece (tp) from multimeric IgM to the C terminus of the human IgG1-Fc and introducing a Leu-to-Cys substitution at position 309 (13, 15). These molecules formed defined multimeric, barrel-shaped structures, typically hexamers, whose binding to receptors was shown to be critically dependent on *N*-linked glycosylation (13, 15). The hexameric Fc also binds the human neonatal receptor (FcRn), an interaction that is known to be critical to the maintenance of a long *in vivo* half-life and to enhanced immunogenicity (13, 21, 22). The efficacy of similar molecules in a mouse model of ITP has been reported in two patent applications (WO2015132364A1 and WO2015132365A1).

Glycosylation is important for correct protein folding in the endoplasmic reticulum and for exporting correctly folded proteins to the Golgi for post-translational modifications (23). Attached glycans also increase the solubility of proteins and have been shown to influence significantly the interactions of IgG with both glycan and Fc receptors (23). Glycosylation of the only available carbohydrate attachment site (Asn-297) in the Fc is essential for interactions with both type 1 and 2 receptors (13, 24, 25). The Fc glycans at Asn-297 are typically biantennary complex types, exhibiting high levels of fucosylation of the core GlcNAc residue, partial galactosylation, and bisecting GlcNac. Of these structures, less than 20% are sialylated (23). The low levels of branching and terminal structures, such as sialic acid, are believed to result from constraints on Asn-297 glycan processing imposed by the Fc protein backbone (23).

The anti-inflammatory properties of the Fc are lost after deglycosylation of IVIG (8, 26, 27), and a population of IgG-bearing α2,6-sialylated Fcs has been identified as making a significant contribution to the control of inflammation in animal models (26, 27). Higher levels of sialylation also lead to longer serum retention times (28, 29). Indeed, the efficacy of sialylated Fc has generated an incentive to modify the existing glycans on Asn-297, either by chemical means or through mutagenesis programs in the Fc protein backbone that disrupt the protein–Asn-297 carbohydrate interface (30–32).

**Figure 1.**
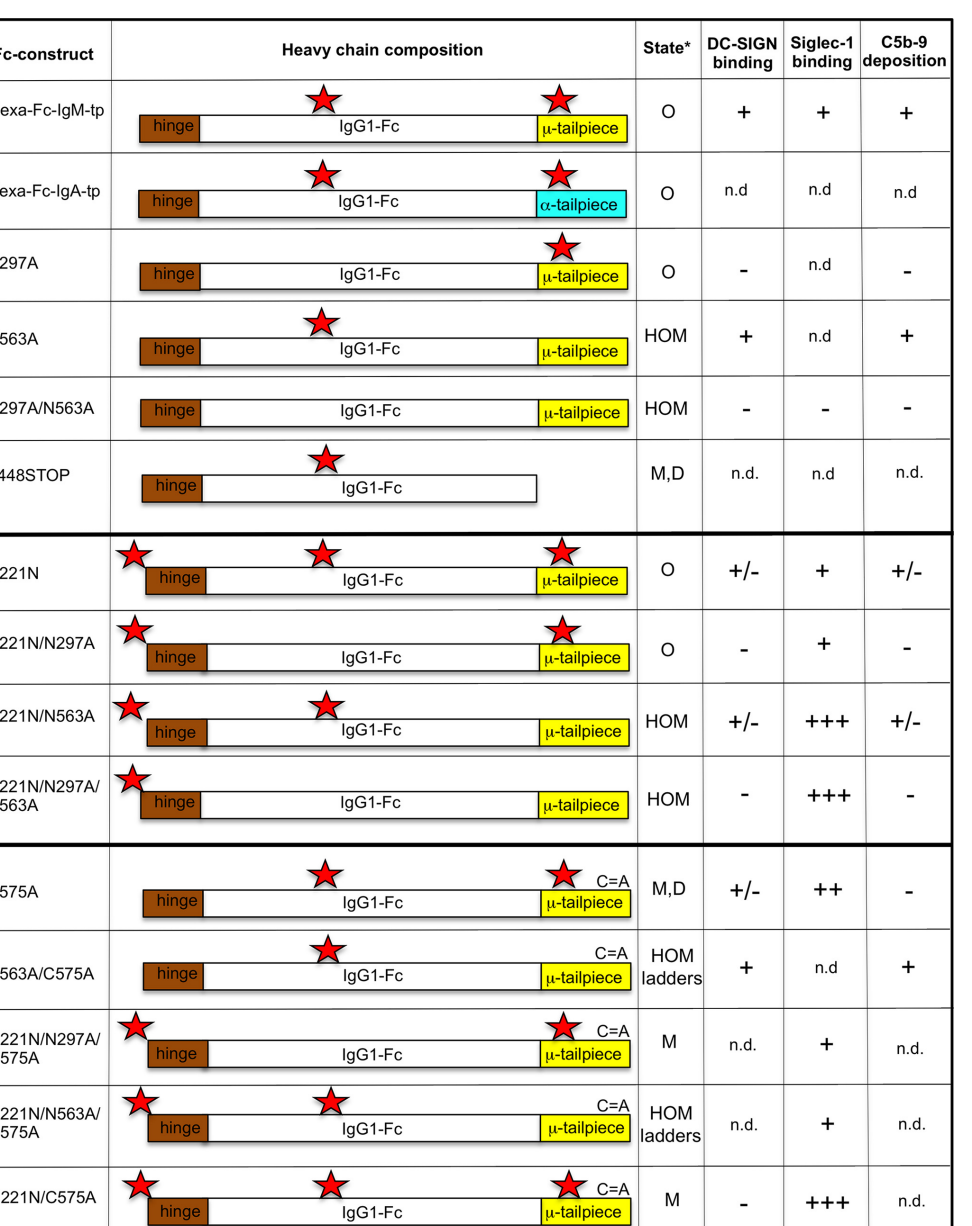
**Schematic showing the glycan and cysteine mutants generated on the hexa-Fc template plasmid hIgG1-Fc-CL309/310CH-TP (13).** *Red stars* indicate the hinge Asn-221, the Cγ2 Asn-297, and the tail piece Asn-563 glycan sites. *C*=*A* indicates mutation of cysteine 575 to alanine in the tail piece. *M*, monomer; *D*, dimer; *O*, oligomer; *HOM*, high-order multimer as determined by size-exclusion analysis and SDS-PAGE; *n.d.*, not determined.

Here we take an unexplored approach to modifying glycosylation by introducing, in various combinations, up to three additional *N*-linked glycosylation sites into exposed areas of the IgG1-Fc fragment (see Fig. 1). Hexa-Fc typically contains two *N*-linked glycosylation sites at Asn-297 in the Cγ2 domain and at Asn-563 in the 18-amino acid IgM tail piece of hexa-Fc (1). We show, for the first time, that it is possible to add a further *N*-linked glycan onto the N terminus of the IgG1-Fc hinge to generate a panel of hypersialylated molecules (the D221N series of mutants) that are still capable of forming multimers that then bind to prototypic sialic acid-dependent receptors, including Siglec-1 (sialoadhesin) and Siglec-4 (myelin-associated glycoprotein). By further mutagenesis of the tail piece Cys-575 to alanine, sialylated multimers can be converted into sialylated monomers that retain strong binding to Siglec-1 and Siglec-4. This study clarifies the role of multiple *N*-linked glycans in maintaining a functional Fc structure and provides routes to the development of antibody therapeutics with bespoke effector functions.

## Results

### Glycosylation influences the multimerization state of hexa-Fc

To determine the contribution of two *N*-linked glycans in hexa-Fc to multimerization and receptor binding, we created a panel of glycosylation mutants by site-directed mutagenesis using the previously described hexa-Fc as the template (Fig. 1) (13, 15). We also inserted an additional *N*-linked attachment site at the N terminus (D221N) to investigate the impact of additional glycosylation on hexa-Fc function (Fig. 1).

**Figure 2.**
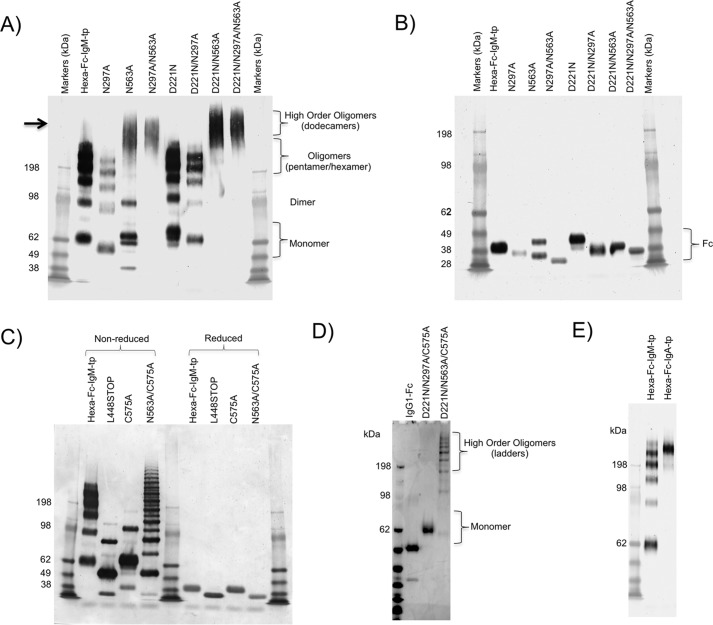
**Characterization of mutant Fc-proteins by SDS-PAGE.** *A*, hexa-Fc, N297A, N563A, and N297A/N563A mutants run as high molecular mass multimers of varying valence under non-reducing conditions. The loss of the N297A glycan does not prevent multimerization but results in lower molecular mass multimers commensurate with the loss of glycans from Asn-297 as seen previously (13). The N563A and N297A/N563A mutants run at molecular masses that approximate to dodecamers (also supplemental Fig. S1). The addition of a N*X*(T/S) glycan sequon to these mutants to generate N-terminally glycosylated hinges (the D221N series of mutants) did not affect multimerization but increased the molecular mass of all mutants and clearly shows that additional sugars may be attached to the N terminus of the IgG1 hinge. *B*, the same mutants as in *A* but run under reducing conditions. The decreasing molecular masses seen in the Fc represent sequential loss of *N*-linked glycans. Thus the N297A/N563A mutant has the smallest molecular mass because it has no glycans attached to the Fc, and D221N has the largest molecular mass because it has three glycans attached. This panel also shows the comparative sizes of the glycans, the Asn-221 and Asn-563 glycans being larger than those attached to Asn-297 (see also mass spectrometry data in Fig. 3 and Fig. S1). Loss of the N563A carbohydrate resulted in two observable Fc fragments that may represent differential glycosylation of Asn-297 or represent some other post-translational modification or proteolytic degradation of this mutant. *C*, the N563A/C575A mutant results in proteins that run as laddered multimers under non-reducing conditions, whereas C575A and the L448STOP mutants run principally as monomers with a small proportion of dimer species observed. *D*, the D221N/N297A/C575A variant runs as a monomer, whereas the D221N/N563A/C575A mutant runs as a ladder of varying molecular masses as seen with the N563A/C575A variant in *C. E*, replacing the 18-amino acid tail piece from IgM with that from IgA resulted in a homogeneous preparation of multimers composed almost entirely of hexamers. All proteins were run under either non-reducing or reducing conditions at 1 μg protein/lane of a 4–8% acrylamide gradient gel, transferred to nitrocellulose, and blotted with anti-human IgG-Fc (Sigma).

Following transfection of these mutated IgG1-Fc DNAs into CHO-K1 cells, stable clonal cell lines were established, and the secreted Fcs were purified by protein A/G affinity chromatography (15). The purified IgG1-Fc proteins were analyzed by SDS-PAGE and immunoblotting with anti-human IgG-Fc (Fig. 2). When analyzed under non-reducing conditions (Fig. 2*A*), the hexa-Fc migrated as monomers and multimers, corresponding to tetramers, pentamers, and hexamers as described previously (13, 15). The N297A mutant resulted in a slight lowering of the molecular mass of all these multimeric forms commensurate with the loss of the glycan at Asn-297 (supplemental Fig. S1*D*) and as described previously (13). Therefore Asn-297 does not contribute to multimerization.

Because removal of the tail piece glycan (Asn-563) in IgM has been shown to enhance multimer formation, mostly as an increase in hexamers over pentamers, we reasoned that a similar mutation introduced into hexa-Fc would also lead to enhanced hexamer formation (33). Removal of Asn-563, as in the N563A, N297A/N563A, D221/N563A, and D221/N297A/N563A mutants, led to the formation of higher-order multimers whose molecular mass (∼650–700 kDa) corresponded to dodecameric forms by size-exclusion chromatography (Fig. 2*A*, *arrow*, and supplemental Fig. S2 for N563A). The type of multimers produced were unaffected by the addition of glycans at Asn-221 (D221N), with all the molecular masses for the D221N molecules being larger than molecules in which Asn-221 was absent (Fig. 2, *A* and *B*).

**Figure 3.**
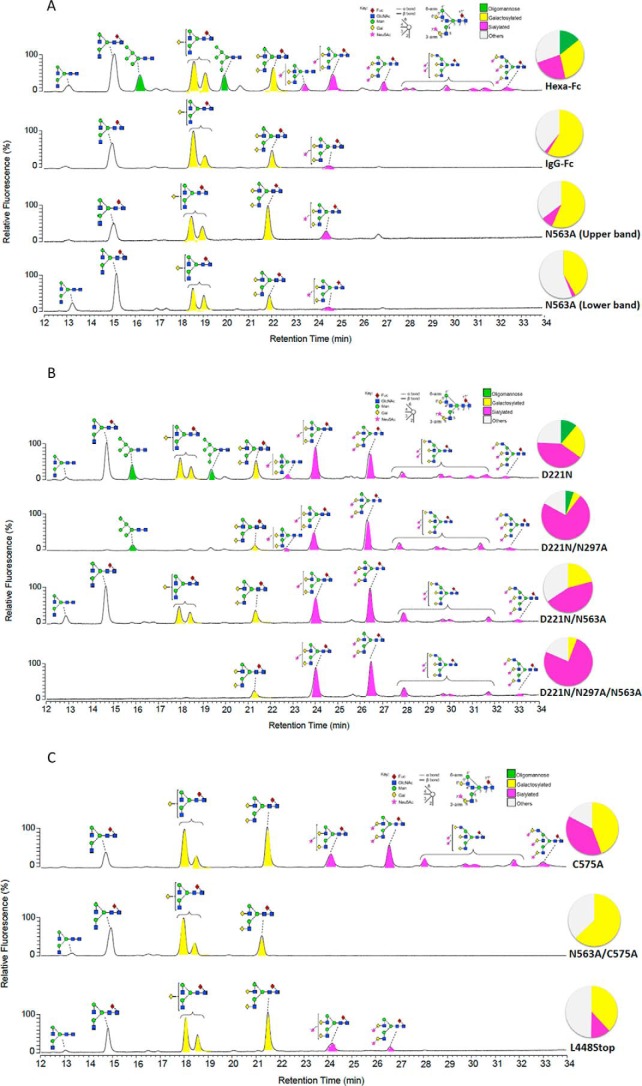
**HILIC-UPLC analysis of 2-AA–labeled *N*-linked glycans from IgG1-Fc mutants expressed by CHO-K1 cells (see Fig. 1).** Normal phase HILIC-UPLC analysis of 2-AA–labeled *N*-linked glycans released from target antibody glycoforms by in-gel protein PNGase F digestion. Glycan profiles for the following variants are shown: hexa-Fc, IgG1-Fc, N563A (upper gel band), and N563A (lower gel band) (*A*); D221N, D221N/N297A, D221N/N563A, and D221N/N297A/N563A (*B*); and C575A, N563A/C575A, and L448STOP (*C*). The *y* axis displays relative fluorescence, and the *x* axis the relative elution time. Inserted pie charts represent the means of two analytical replicates; the pie charts summarize the quantification of oligomannose-type (*green*), galactosylated (*yellow*), and sialylated glycans (*pink*) on individual sites. Quantifications are based on the peak lists in supplemental Fig. S1 and supplemental Table S1. Percentages corresponding to this figure can be found in Table 1.

By running these mutants under reducing conditions, we were able to determine the relative sizes and occupancy of the various glycans attached at each position, showing that the Asn-221 and Asn-563 attached glycans are larger than those attached to Asn-297 (Fig. 2*B*). These observations on the molecular masses of the various glycoforms were also confirmed by hydrophilic interaction chromatography (HILIC)-UPLC analysis of the carbohydrates as described below (Fig. 3 and supplemental Fig. S1).

### N-Linked glycoprofiling of hexa-Fc proteins

Glycans were released from purified Fc constructs via protein *N*-glycosidase F (PNGase F). The free sugars were fluorescently labeled and resolved via HILIC using the ACQUITY® UPLC ethylene bridged hybrid amide column. The HILIC-UPLC spectra from the Fc mutants expressed in CHO-K1 cells are shown in Fig. 3.

The glycans from IgG1-Fc are composed of a series of fucosylated, biantennary, complex-type carbohydrates, typical of the protein-directed glycosylation observed for IgG (Fig. 3*A*). The most abundant species observed were galactosylated structures, a very small population (∼2%) of sialylated material, and a complete absence of oligomannose structures (Table 1), findings that are broadly consistent with previous observations (13). In contrast, hexa-Fc displayed a 2-fold reduction in galactosylated sugars and enhanced oligomannose-type (Man_5_GlcNAc_2_, Man_6_GlcNAc_2_) structures, consistent with a previous observation for their putative contribution to DC-SIGN binding (13) (Fig. 3*A* and Table 1). The loss of Man_5_GlcNAc_2_ and Man_6_GlcNAc_2_ structures in the N563A and D221N/N563A multimers show that these oligomannose structures are attached at Asn-563 in the tail piece and not at Asn-297 as previously modeled (13).

**Table 1:** **Glycan composition expressed as percentages of the total for hexa-Fc variants shown in Fig. 1**

Samples	Glycan composition		
	Oligomannose	Galactosylated	Sialylated
	%		
Hexa-Fc	14.4	32.1	23.2
IgG1-Fc		58.6	2.3
N563A (upper band)		56.5	8.1
N563A (lower band)		41.6	2.5
D221N	11.1	23.8	40.9
D221N/N297A	5.3	5.1	72.6
D221N/N563A		20.9	44.8
D221N/N297A/N563A		5.8	75.7
C575A		44.3	38.4
N563A/C575A		62.9	
L448STOP		38.1	12.2

Triantennary species, not normally observed on the Fc, were detected on hexa-Fc (Fig. 3*A* and supplemental Fig. S1 and Table S1). Additionally, increased terminal sialylation was also prominent on the hexa-Fc. Unusual di- and trigalactosylated and di- and trisialylated species were also detected in the HILIC-UPLC spectra of hexa-Fc. Similar unusually sialylated structures have been detected in mouse serum glycoproteins, and all are attached via α2,3 linkages, as expected for proteins expressed by CHO-K1 cells (34). The structural assignments were confirmed by electrospray mass spectrometry for all the recombinant Fc proteins (supplemental Fig. S1 and Table S1). The loss of these sialylated structures in the N563A mutant shows that these complex structures must be located on the tail piece Asn-563 glycan in hexa-Fc (Fig. 3*A*). Under reducing conditions, the N563A mutant appeared as two separate bands. *N*-Linked glycan analyses of these two bands revealed them to contain similar glycoprofiles but in different proportions (Fig. 3*A*).

We next generated the novel D221N series of mutants to investigate whether *N*-linked sugars could be attached to the exposed N terminus of the hinge and what the impact of such glycosylation would be on glycan processing at Asn-297 and Asn-563 (Fig. 1). The addition of D221N onto the hexa-Fc scaffold doubled the overall sialic acid content while reducing the oligomannose-type glycans (Table 1 and Fig. 3*B*). The D221N mutation was clearly the main driver for extensive sialylation, because the removal of both Asn-297 and Asn-563 in the D221N/N297A/N563A mutant resulted in recombinant multimers whose glycan composition was 75% sialylated with complete loss of oligomannose and a 6-fold reduction in galactosylated glycans that would normally be located on Asn-297 in the hexa-Fc (Fig. 3*B* and Table 1). As expected, no glycans could be detected on the glycosylation-deficient double mutant N297A/N563A, and only weak signals that could not be assigned specific structures were observed for the N297A mutant (supplemental Fig. S1*D*).

### The Asn-297 glycan is critical for interactions of hexa-Fc with DC-SIGN but not Siglec-1

**Figure 4.**
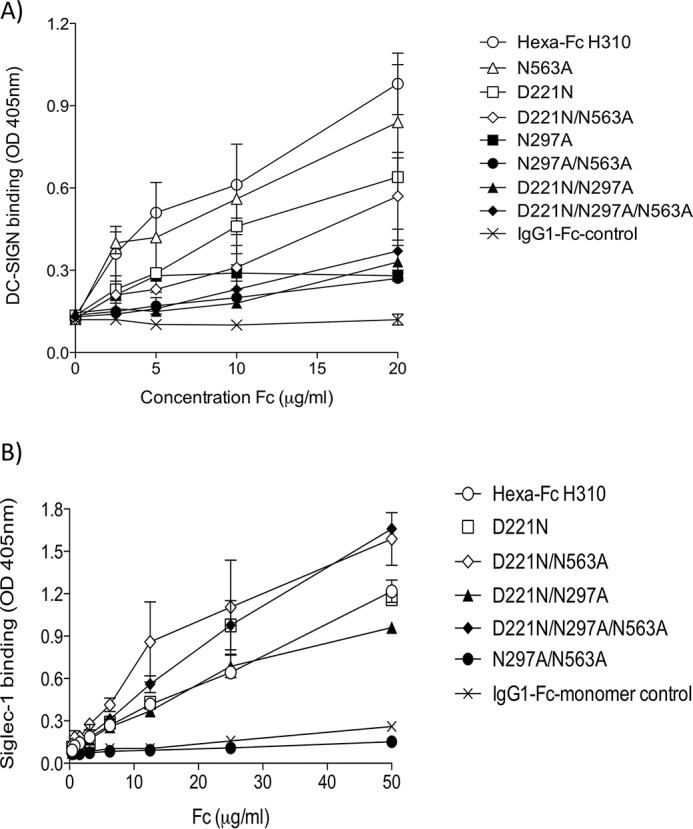
**Binding of IgG1-Fc variants to glycan receptors.** *A*, mutants lacking the Asn-297 glycan are severely restricted in their capacity to bind DC-SIGN by ELISA. The addition of an *N*-linked sugar at position 221 results in proteins with a reduced capacity to bind DC-SIGN compared with their equivalent variants in which Asn-221 is absent. *B*, the hypersialylated D221N mutants bind Siglec-1. No binding was observed with the N297A/N563A glycan-deficient mutant (*error bars* represent standard deviations around the mean value, *n* = 2 independent experiments).

To determine which *N*-linked glycan on the hexa-Fc contributes to receptor binding, we investigated the interaction of the panel of *N*-glycosylation mutants with soluble recombinant tetrameric human DC-SIGN by ELISA (Fig. 4*A*). As previously published, hexa-Fc bound DC-SIGN (13, 15). Removal of Asn-297 resulted in a dramatic loss of binding to this receptor, whereas removal of Asn-563 (as in the N563A mutant) had only a minor effect (Fig. 4*A*). The loss of oligomannose type sugars (Man_5_GlcNAc_2_ and Man_6_GlcNAc_2_) in the N563A mutant (Fig. 3*A*) that still binds DC-SIGN highlights that oligomannose structures are not necessary for DC-SIGN interactions by multimers and that other glycan structures found at Asn-297 are involved. A similar important contribution of the Asn-297 glycan to DC-SIGN binding was seen with the D221N series of mutants, which all possessed reduced interactions with DC-SIGN compared with the controls that lack the D221N insertion (Fig. 4*A*). This shows that the presence of the *N*-linked glycan at Asn-221 can negatively affect interactions mediated via the Asn-297 glycan. The lack of binding to DC-SIGN by both the D221N/N297A and D221N/N297A/N563A mutants, whose glycans are respectively 73 and 75% sialylated, also shows that α2,3-linked sialic acid containing structures do not make a significant contribution to human DC-SIGN binding while confirming the critical role of Asn-297 to binding.

Although we tentatively suggested that oligomannose may make a contribution to DC-SIGN binding by the hexa-Fc (14% oligomannose) (13), the requirement for oligomannose in DC-SIGN binding is clearly not essential, because the N563A and N563A/C575A mutants that are both devoid of oligomannose can still bind DC-SIGN (Fig. 3, Table 1, and supplemental Fig. S1 and Table S1), although not as well as hexa-Fc (Fig. 4*A*). The data from these two mutants, whose glycosylation profiles were very similar to monomeric IgG1-Fc, show that glycan structures other than oligomannose on Asn-297 can contribute to DC-SIGN binding (Fig. 2*A*). This finding may also provide a rational explanation for our previous conflicting observation that endoglycosidase H treatment of hexa-Fc did not abrogate DC-SIGN binding (13).

The remarkable sialylation profile of the D221N series of mutants (Fig. 3*B*, Table 1, and supplemental Fig. S1) led us to investigate interactions with the prototypic sialic acid-dependent human receptor Siglec-1 (Fig. 4*B*). Human Siglec-1, also known as sialoadhesin or CD169, is a cell surface receptor restricted to monocytes and macrophages with a predilection for α2,3 glycosidic linkages. All the D221N panel of Fc proteins bound Siglec-1 irrespective of the presence or absence of either Asn-297 or Asn-563 (Fig. 4*B*). Indeed binding by the D221N/N297A/N563A mutant shows that Asn-221 is sufficient for this interaction with Siglec-1 to occur. As expected, the complete absence of carbohydrate (as found in the N297A/N563A double knock-out) or the absence of sialic acid-containing glycans (as in the IgG1-Fc monomer) led to proteins that are unable to bind Siglec-1 (Fig. 4*B*). We have also investigated binding to Siglec-2 (CD22), a receptor that has a binding preference for α2,6-glycosidic linkages, and observed low binding of these α2,3-linked sialo-Fcs to Siglec-2 (data not shown).

### The Asn-297 glycan is critical for interactions of hexa-Fc with the classical Fcγ receptors and complement

**Figure 5.**
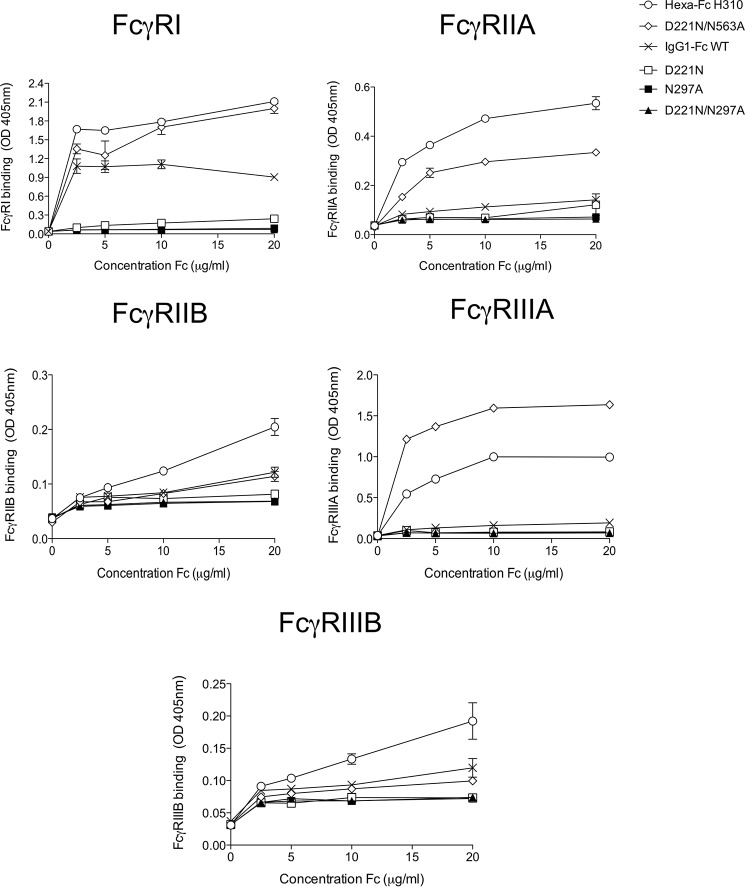
**Binding of *N*-linked glycan mutants to classical FcγRs assessed by ELISA.** Removal of the Asn-297 glycan in the N297A and D221N/N297A mutants resulted in a dramatic loss of binding to all FcγRs. The presence of the N-terminal hinge glycan (D221N) also reduced binding to all FcγRs, although this loss of binding could be reversed with the simultaneous loss of Asn-563 (D221N/N563A mutant). A marked improvement of binding to FcγRIIIA was observed with this D221N/N563A mutant (*error bars* represent standard deviations around the mean value, *n* = 2 independent experiments).

We next investigated which of the *N*-linked glycans on the hexa-Fc contributes to Fcγ receptor (FcγRs) binding (Fig. 5). As previously published, hexa-Fc bound with avidity and specificity to all the human FcγRs investigated (13). Removal of Asn-297 in either the N297A or D221N/N297A mutants completely abolished binding to all the human FcγRs, demonstrating a clear requirement for this Asn-297 glycan in interactions with FcγRs. Attachment of N-terminal glycans at Asn-221 inhibited binding to all FcγRs, although the removal of N563A in the tail piece reinstated binding of the D221N-containing mutant (D221N/N563A) to FcγRs and in particular to FcγRIIIA. Thus the N563A tail piece glycan is not required for binding to FcγRs.

**Figure 6.**
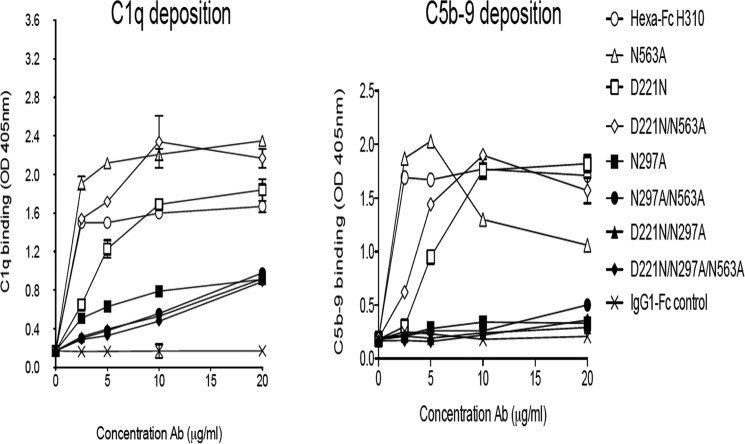
**Binding of *N*-linked glycan variants to complement assessed by ELISA.** Removal of the Asn-297 glycan as in the N297A, N297A/N563A, D221N/N297A, or D221N/N297A/N563A mutants resulted in dramatic loss of binding to both C1q and subsequent C5b-9 deposition. The N563A and D221N/N563A mutants were as good as wild-type hexa-Fc at activating complement. Although unable to bind FcγRs, the D221N mutant was clearly capable of binding C1q, leading to C5b-9 deposition, although not as efficiently as either hexa-Fc or N563A (*error bars* represent standard deviations around the mean value, *n* = 2 independent experiments).

The multimeric structure of hexa-Fc also enables strong activation of the classical complement pathway (13). To investigate which *N*-linked glycan on the hexa-Fc is important for C1q binding and C5b-9 deposition, we screened the panel of mutants by ELISA (Fig. 6). Binding to C1q and subsequent C5b-9 deposition were critically dependent on the presence of the Asn-297 glycan. Removal of the Asn-563 tail piece carbohydrate in either the N563A or D221N/N563A mutants had little effect on complement activation, in stark contrast to all the mutants where Asn-297 was absent (Fig. 6). The addition of an *N*-linked carbohydrate to the N terminus of the hinge (D221N and D221N/N563A) reduced both C1q binding and complement activation, compared with equivalent proteins that lack Asn-221 (Fig. 6). Thus the presence of Asn-297 is essential for complement activation in multimers.

### The 18-amino acid C-terminal tail piece and in particular Cys-575 are critical in the formation of multimeric IgG1-Fc

To investigate the structural features of the human IgM tail piece required for multimerization and function of the hexa-Fc, we generated further mutants, including L448STOP, C575A, N563A/C575A, D221N/N297A/C575A, D221N/N563A/C575A, D221N/C575A, and hexa-Fc-IgA-tp in which the 18-amino acid tail piece from IgM was replaced with that from human IgA (Fig. 1). Deletion of the entire tail piece by stop codon introduction (L448STOP) completely prevented the formation of higher order multimers, although a very small proportion of dimer and other multimers could still be seen (Fig. 2*C*). In the absence of the entire tail piece, the small proportion of multimers and dimer observed can only arise through intermonomeric disulfide bridging at Cys-309 (see Fig. 8). Similarly, substitution of the Cys-575 residue of the tail piece with alanine resulted in the secretion of mostly IgG-Fc monomers, but there is also evidence of a small proportion of higher order multimers (Fig. 2*C*). It is intriguing that the introduction of a glycan at Asp-221 together with the C575A mutation yields only monomers in the presence of Asn-563 (Fig. 2*D*). This shows that the Asn-221 hinge glycan may constrain multimerization mediated either through Cys-309 or the tail piece.

Deletion of both Asn-563 and Cys-575 in the tail piece (N563A/C575A) resulted in a laddering pattern of different molecular masses from ∼50 to greater than 400 kDa (Fig. 2*C*), most likely representing monomers, dimers, trimers, tetramers, pentamers, and hexamers, although molecules as uniform as those seen with the N563A-containing mutants were not observed (Fig. 2*A*). These ladders probably arise through disulfide bond formation between Cys-309 of two adjacent monomers (Fig. 8). The introduction of the C575A mutation onto the backbone of D221N/N297A (to generate the D221N/N297A/C575A mutant) resulted in monomers (Fig. 2*D*), whereas the introduction of C575A onto the D221N/N563A backbone resulted in a similar laddered pattern of multimers (Fig. 2*D*) as seen previously with N563A/C575A (Fig. 2*C*). Replacement of the 18-amino acid tail piece from IgM with that from IgA resulted in a homogeneously multimeric protein, indicating that amino acids other than Asn-563 and Cys-575 in the IgM tail piece are involved in determining the overall valence and quaternary structure of the assembled multimer (Fig. 2*E*).

### Substitution of Cys-575 with Ala generates monomers with altered glycosylation profiles and enhanced binding to glycan receptors

**Figure 7.**
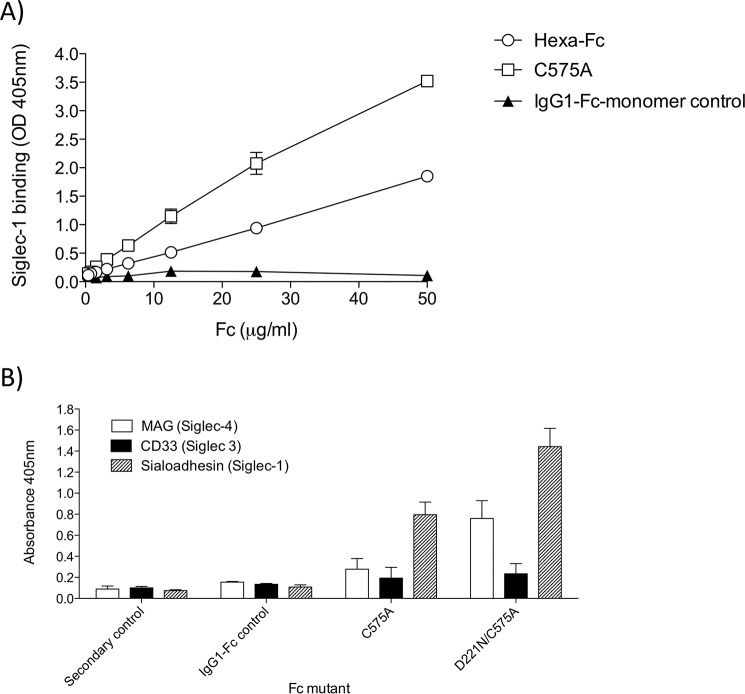
**Binding of monomeric IgG1-Fc glycan variants to sialic acid-binding immunoglobulin-type lectins (Siglecs) with specificity for α2,3-linked sialic acid.** *A*, the C575A monomer binds Siglec-1. *B*, the D221N/C575A monomer binds Siglec-1 and Siglec-4. ELISA as described under “Experimental procedures” with receptors coated down at 2 μg/ml and Fc-fragments at 20 μg/ml in TMS buffer (*error bars* represent standard deviations around the mean value, *n* = 2 independent experiments).

The C575A glycan profile when compared with N563A/C575A shows that the Asn-563 glycan in the tail piece could be sialylated in the C575A monomer (Figs. 2*C* and 3*C*). The C575A glycan profile resembles that seen with complex multimers including hexa-Fc (Fig. 3*C* and Table 1), with approximately 16-fold increase in sialylation compared with the IgG1-Fc control (Table 1). The C575A monomer was shown to be fully competent with respect to Siglec-1 binding (Fig. 7*A*), and binding to all the FcγR was broadly similar to the IgG1-Fc or the L448STOP monomer control (supplemental Fig. S3). In contrast to hexa-Fc, the C575A mutant bound C1q (supplemental Fig. S4*A*) but was unable to activate complement as determined by C5b-9 deposition (supplemental Fig. S4*B*).

Given the marked binding of C575A to Siglec-1 (Fig. 7*A*), we wondered whether monomeric C575A (with two *N*-linked sugars) or monomeric D221N/C575A (with three *N*-linked sugars) could bind other glycan receptors with a known preference for α2,3-linked sialic acid (Fig. 7*B*) (35). Although C575A showed marked binding to Siglec-1, the D221N/C575A monomer showed enhanced binding to both human Siglec-1 and Siglec-4, another glycan receptor with a known preference for α2,3-linked sialic acid. However, not all glycan receptors with a preference for α2,3-linked sialic acid could bind. For example, human Siglec-3 (CD33) was unable to bind either Fc-monomer mutant. We were unable to test binding to human Siglec-5 because we observed significant direct binding of the Fab′_2_ detecting reagent to this receptor. The Fab′_2_-mediated binding to Siglec-5 was dependent on glycans, because treatment of the Fab′_2_ detect with neuraminidase abrogated binding to Siglec-5 (supplemental Fig. S5). Siglec-5 may therefore be a target for Fab glycans that have also been associated with the anti-inflammatory activity of IVIG (36).

## Discussion

We previously demonstrated the importance of carbohydrate in the binding of hexa-Fc to DC-SIGN and in the activation of the complement cascade (13). In this study we used a protein engineering approach to determine the features of hexa-Fc required for multimerization and binding to receptors, by investigating the relative contributions of two *N*-linked glycosylation sites found at Asn-297 in the Cγ2 domain and Asn-563 located in the 18-amino acid IgM tail piece of hexa-Fc (Fig. 1).

Human IgA and IgM antibodies that multimerize differ from other isotypes in possessing an 18-amino acid C-terminal extension of the heavy chain termed the tp, which previous studies have implicated in multimerization of monomer subunits in both IgA (37) and IgM (38). In line with these earlier studies, we found that complete removal of the tail piece from hexa-Fc, as in the L448STOP mutant, resulted in proteins that were mostly monomeric, although a very small proportion of dimers was observed (Fig. 2*C*). Furthermore, attachment of the 18-amino acid IgA tail piece, rather than the IgM tail piece, resulted in a homogeneous preparation of multimers with no monomers, dimers, or other lower order multimeric forms being detectable (Fig. 2*E*).

The removal of the tail piece carbohydrate at Asn-563 has been shown to enhance multimer formation in IgM (33) while reducing multimerization in IgA (37, 39). We therefore wondered what impact the removal of Asn-563 would have on hexa-Fc containing the IgG1-Fc backbone and IgM tail piece. Remarkably, greater than 95% of proteins from such mutants deficient in Asn-563 were secreted with a molecular mass of ∼600 kDa, approximating to dodecamers (Fig. 2*A* and supplemental Fig. S2) (33). There is a precedent for dodecamer formation when the 18-amino acid tail piece from IgA was fused to the C terminus of CD4, although whether dodecamers could arise when the IgM tail piece is fused to the human IgG1-Fc has not been documented previously (40).

**Figure 8.**
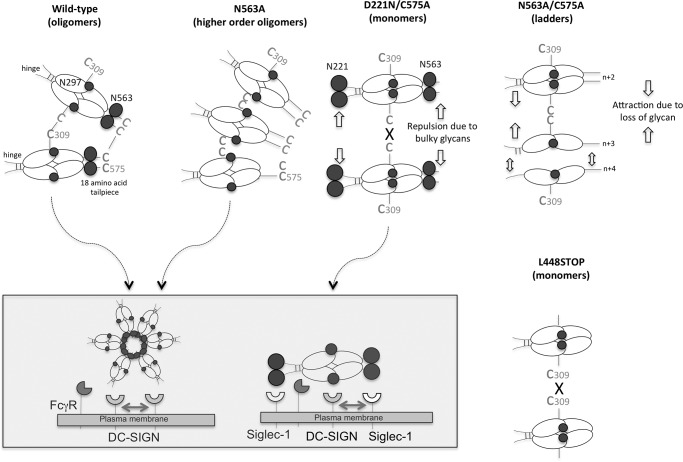
**Model showing the contribution of different *N*-linked glycan and cysteine residues on Fc stoichiometry.** The presence of Cys-575 allows optimal disulfide bonding between tail pieces of monomeric-Fcs. The tail piece glycan Asn-563 controls the number of monomeric tails that fit into the central corona (five to six in the case of hexa-Fc) while still allowing Cys-309 interdisulfide bridge formation. Cys-575 allows disulfide bonding between tail pieces of different monomers, but the absence of the Asn-563 glycan (the N563A mutant) allows many more tail pieces (up to twelve in the case of dodecamers) to fit into the central corona while still allowing disulfide bond formation through Cys-309 and/or Cys-575. The absence of Cys-575 prevents disulfide bonding between tail pieces, thereby generating sialylated monomers at Asn-563. The additional Asn-563 tail piece glycan in these monomers must explain the increased binding seen to Siglec-1 (Fig. 3, *A* and *B*, and *inset* in this figure). The bulkier Asn-563 glycan with its predicted overall negative charge may lead to repulsion between two monomers, thus preventing disulfide bond formation between two Cys-309 residues in each monomeric Fc. The loss of both Asn-563 and Cys-575 (the N563A/C575A mutant) means that the observed laddered multimers must arise through Cys-309–mediated disulfide bonding in the Cγ2 domain. The presence of monomers, dimers, trimers, tetramers, pentamers, hexamers, and other intermediates in this mutant (Fig. 2*C*) suggests that these structures arise through a different mechanism, most likely via the sequential addition of 25-kDa half-mer Fc units at Cys-309. The lack of observable ladders with the L448STOP mutant implies that other amino acids in the tail piece are involved in bringing about monomer interactions that then facilitate disulfide bonding through either Cys-309 and/or Cys-575. Monomers with glycans located at both the N- and C-terminal ends of the Fc (Asn-221 and Asn-563) may allow for binding to receptors in *cis* as shown (*inset*).

In contrast to hexa-Fc, the formation of native dodecameric IgM, IgG, IgE, or IgA is unlikely given additional constraints imposed by the size of the Fc (extra Cμ2 domain in the Fc of IgM and IgE) or the associated F(ab)_2_ arms in each monomer of the heavy chains of these antibody types. Therefore the lack of bulky carbohydrates in the tail piece—the absence of both Fab domains and the extra C1 constant domain in the Fc of IgM or IgE—allows more of the unstructured tail pieces in the N563A or D221N/N563A mutants to form intermonomeric disulfide bonds via Cys-575, thus allowing for the formation of higher ordered multimers over other multimeric species described previously (Fig. 8). Despite their increased valence, no improvement in the ability of either the N563A or D221N/N563A mutants to bind DC-SIGN or activate complement were observed (Figs. 4*A* and 6). Furthermore, the N563A, D221N/N563A, and the N563A/C575A mutants all show that binding to DC-SIGN is totally dependent on the presence of Asn-297. These mutants may therefore have beneficial utility in various therapeutic applications where enhanced valence is required at no cost to receptor binding or complement activation, for example in the delivery of more copies of antigen in vaccine applications (1).

This study also expands our knowledge of glycosylation on Fc activity. Under normal circumstances, a single *N*-linked glycosylation site exists at amino acid 297 in the Cγ2 domain of all IgG subclasses (41) that we and others have shown is critical for interactions with FcγRs and DC-SIGN (13, 27). We therefore hypothesized that the addition of an extra *N*-linked carbohydrate onto an exposed region of the Fc would enhance interactions with glycan receptors. We therefore engineered an additional *N*-linked sequon at position 1 of the Fc polypeptide chain to produce the D221N series of mutants (Fig. 1). We show for the first time that it is possible to add *N*-linked glycans to the N terminus of the hinge of IgG1-Fc to generate molecules that are still capable of forming multimers (Fig. 2*A*). This was unanticipated, because *N*-linked glycans are not typically attached to the hinges of native IgG molecules (or of other classes of antibody), because they are presumed to interfere with disulfide bond formation and the capacity of the hinge to act as a flexible linker. Native antibodies such as IgA likely *O-*glycosylate their hinges for this reason.

Despite containing larger, more complex glycans (Figs. 2 and 3, Table 1, and supplemental Fig. S1), no improvement in binding to either DC-SIGN (Fig. 4*A*) or C1q (Fig. 6*A*) over hexa-Fc was observed with the D221N panel of mutants. The presence of the introduced glycan at Asn-221 appears mostly to have a detrimental effect on FcγR binding, presumably by interfering with the FcγR binding site located within the lower hinge region (41). The Asn-221 attached glycans are larger than those found at Asn-297 (Fig. 3 and supplemental Fig. S1), and therefore, as already shown with multimeric Fc fusions to antigens, their presence may interfere with FcγR binding (15). Although this may be the case for D221N hexamers, it clearly does not hold for the D221N/N563A, which had markedly improved binding to FcγRIIIA (Fig. 5). We do not yet know the structure of the higher-order multimers, but these data might anticipate significant yet subtle differences in their structure compared with hexa-Fc.

Because removal of the tail piece *in toto* (the L448STOP mutant) resulted in the formation of a small proportion of dimers (Fig. 2*C*), presumably through intermonomer disulfide bridges via Cys-309 in the Cγ2 domain of hexa-Fc, we engineered two further tail piece mutants in the presence or absence of Asn-563 to explore the role of the tail piece Cys-575 in multimerization and receptor binding (Fig. 1). Removal of Cys-575 without loss of the Asn-563 glycan resulted in molecules that mostly formed sialylated monomers (Figs. 2*C* and 3*C*). The monomeric C575A mutant could bind Siglec-1 (Fig. 7) and was comparable with the D221N mutant in respect of DC-SIGN binding; however, the C575A monomer was still able to bind FcγRs and, like the IgG1-Fc control monomer, was unable to activate complement (supplemental Fig. S4).

The presence of the Asn-563 glycan in the absence of disulfide-mediated multimerization through Cys-575 presumably restrains further disulfide bonding via Cys-309 (Fig. 8), thus favoring the formation of monomers and allowing for interactions with glycan receptors such as Siglec-1 in the absence of complement activation (Fig. 7 and supplemental Fig. S4). Surprisingly, removal of both Asn-563 and Cys-575 still allowed for the formation of multimers of various molecular masses that, in the absence of any other free cysteines, must arise through Cys-309 (Fig. 8). In the case of the N563A/C575A multimers, all the binding to DC-SIGN is now due to interactions via glycans attached to Asn-297. In the absence of both Cys-575 and Asn-563, other amino acids within the 18-amino acid tail piece must allow for interactions between individual Fc monomers that then allow disulfide bond formation via Cys-309 (Fig. 8), which cannot occur with the L448STOP mutant, in which the whole tail piece was removed. The hypothesis that other tail piece residues, other than Asn-563 and Cys-575, are involved in recruiting monomer-monomer interactions that permit the final quaternary structure of hexa-Fc to form is supported by the finding that the use of the IgA tail piece instead of that from IgM leads to improved multimerization and yields of hexameric IgG1-Fc (Fig. 2*E*).

Taken together, our results show that the Asn-563 tail piece glycan serves as a spacer, limiting to five or six the number of monomeric IgG1-Fc subunits that can be incorporated into an multimer (Fig. 8). As multimers, binding to glycan receptors is entirely dependent on glycans attached at Asn-297 or Asn-221, because the glycans at Asn-563 are buried in multimers, only becoming available to influence receptor interactions when found in the context of monomeric IgG1-Fcs, such as the C575A or D221N/C575A mutants.

IgG-Fc sialylation has emerged as an important but controversial concept for regulating anti-inflammatory activity of antibodies (6). Translating this concept to potent anti-inflammatory therapies has been hampered by the difficulty of generating suitably enriched sialylated products for human use. All approaches to date have focused on chemical or genetic modifications to the only available *N*-linked glycan found at position Asn-297 in the Fc (30). We describe two complementary approaches to increasing the sialic acid content of the Fc, first by insertion of the 18-amino acid tail piece from IgM onto the C terminus of the IgG1-Fc into which a cysteine-to-alanine substitution is made at Cys-575 (Fig. 3 and Table 1) and second by the addition of an extra *N*-glycan at Asn-221. This D221N approach results in significantly higher sialylation over C575A, which also led to improved binding to both Siglec-1 and Siglec-4. Monomers in which all three glycosylation sites (Asn-221, Asn-297, and Asn-563) are sialylated *e.g.* D221N/C575A, may therefore yield molecules with greater efficacy for use in sialic acid-dependent therapies. This approach requires no expensive *in vitro* enzymatic or complex chemical modifications of the Fc glycan and no requirement for glycosidase deficient/transgenic cell lines for their manufacture.

In contrast with the Asn-297 glycan, which is largely buried within the Fc cavity, both Asn-221 and Asn-563 are located at the N- and C-terminal tips of the Fc and, as our data show, would therefore be more accessible for post-translational modifications by glycan-modifying enzymes. Although C-terminal tail piece sialylation in monomers such as the C575A mutant may appear attractive for therapy, we have recently observed that C-terminal tail piece additions can favor interactions with other plasma proteins (42), and therefore hinge-focused approaches to enhancing sialylation (as in D221N mutants) may be more tractable for therapeutic development.

Generating commercial multimeric Fcs raises significant bioprocessing and safety issues that are not found with monomeric Fc production. For example, high-mannose type glycans found in hexa-Fc have been shown to increase IgG clearance rates because of cellular uptake via the mannose receptor (43). Recombinant monomeric Fcs developed here that are devoid of oligomannose and yet show improved binding to selected glycan receptors may therefore have significant therapeutic potential, for example as replacements for IVIG (13, 30). Furthermore, given the known effects of Fc sialylation in reducing IgG antibody-dependent cellular cytotoxicity activity (44), it may also be possible to use the D221N/C575A mutations to develop therapeutic antibodies with modified effector functions.

Multimeric Fcs may nonetheless be useful, for example when delivering antigens in vaccines or as high avidity receptor blockers. Many pathogens rely on glycans to infect host cells (45), and differentially glycosylated Fc multimers may be useful inhibitors of infection. One immediate application for our hypersialylated molecules may be to block Siglec-1–dependent trans-infection of lymphocytes by retroviruses, including HIV and human T-cell leukemia viruses (46). We anticipate that expression of these mutants in human cell lines, *e.g.* HEK, will bestow hypersialylated molecules with α2,6 linkages with improved binding to α2,6-dependent receptors like Siglec-2 that are implicated in IVIG efficacy (14). Such receptor mimicry strategies need to overcome the high avidity of the natural receptor generated by the sum of the multiple low-affinity glycan binding sites that may now be achievable with the D221N series of hypersialylated multimers. Thus, by adding or removing glycosylation and disulfide bonding sites within hexa-Fc, new portfolios of effector functions can be generated.

## Experimental procedures

### Production of glycosylation mutants

The generation of hexa-Fc has been previously described (13, 15). The following mutants were constructed by PCR overlap extension mutagenesis from the wild-type vector (pFUSE-hIgG1-Fc-TP-LH309/310CL) as the template, using pairs of internal mismatched primers for each mutant as follows: N297A, 5′-GAGCAGTACGCCAGCACGTAC-3′/3′-CTCGTCATGCGGTCGTGCATG-5′; N563A, 5′-CCCTGTACGCCGTGTCCCTG-3′/3′-GGGACATGCGGCACAGGGAC-5′; D221N, 5′-GTTAGATCTAACAAAACTCAC-3′/3′-CAATCTAGATTGTTTTGAGTG-5′; L450STOP, 5′-TCTCCGGGTAAATGAGTCCTAGGACCC-3′/3′-AGAGGCCCATTTACTCAGGATCCTGGG-5′; C575A, 5′-ACCCTGCTTGCTCAACTCT-3′/3′-GGCCAGCTAGCTCAGTAGGCGGTGCCAGC-5′; N297A/N563A, primer pair N563A was used on the N297A mutant plasmid; D221N/N297A, primer pair N297A was used on D221N mutant plasmid; D221N/N563A, primer pair N563A was used on the D221N mutant plasmid; D221N/N297A/N563A, primer pair N563A was used on the D221N/N297A mutant plasmid; and N563A/C575A, primer pair C575A was used on the N563A, D221N, D221N/N563A, or D221N/N297A mutant plasmids. The following flanking primers were used in the overlap PCR. These are 5′-ACCCTGCTTGCTCAACTCT-3′ and 3′-TGGTTTGTCCAAACTCATCAA-5′, which are 71 or 22 base pairs upstream or downstream of the EcoRI or NheI (all from New England Biolabs) sites used in subcloning into the wild-type vector. DNA coding for the human IgA tail piece (PTHVNVSVVMAEVDGTCY) was synthesized by EUROFINS and cloned as an AvrII/NheI fragment into pFUSE-hIgG1-Fc-TP-LH309/310CL. To verify incorporation of the desired mutation and to check for PCR-induced errors, the entire coding sequence of the new expression plasmids were sequenced on both strands using the same set of flanking primers (Sanger Sequencing Service, Source Bioscience). CHO-K1 cells (European Collection of Cell Cultures) were transfected with plasmid using FuGENE (Promega), and positive clones were selected, expanded, and purified as previously described for hexa-Fc (13, 15).

### Enzymatic release of N-linked glycans

Recombinant proteins (50 μg) were fractionated by SDS-PAGE on Novex® NuPAGE Bis-Tris 4–12% precast gels (Life Technologies) under reducing condition. After staining with Coomassie Blue, gel bands were excised, washed five times with alternating acetonitrile and water, and air-dried. Each gel band was rehydrated in a reaction buffer (250 μl of 50 mm NaHCO_3_, pH 7.4) containing 500 units/ml PNGase F (New England Biolabs) and incubated at 37 °C for 16 h. The released glycans were extracted from the gel matrix by washing three times with water and then dried in a SpeedVac Concentrator Plus (Eppendorf).

### Fluorescent labeling of N-linked glycans

PNGase F-released glycans were fluorescently labeled with 2-aminobenzoic acid (2-AA) as previously described (31). Briefly, glycans were resuspended in 30 μl of water, followed by the addition of 80 μl of labeling mixture (3% (w/v) 2-AA, 4.5% w/v sodium cyanoborohydride, 4% (w/v) sodium acetate trihydrate, and 2% w/v boric acid in methanol). After incubation at 80 °C for 1 h, samples were diluted with 1 ml of 97% (v/v) acetonitrile before being loaded onto Speed Amide-2 cartridges (Applied Separations) and eluted with 2 ml of water to remove excess label.

### Exoglycosidase sequencing of N-linked glycans

The 2-AA–labeled glycans were sequentially digested using the following exoglycosidases: α2–3,6,8 neuraminidase from *Clostridium perfringens* (New England Biolabs), β1,4-galactosidase from *Bacteroides fragilis* (New England Biolabs), α-l-fucosidase from bovine kidney (Sigma-Aldrich), β-*N*-acetylglucosaminidase from *Xanthomonas manihotis* (New England Biolabs), and α(1–2,3,6)-mannosidase from jack bean (Sigma-Aldrich). Endoglycosidase H from *Streptomyces picatus* (New England Biolabs) was used for quantification of oligomannose structures. Digestions were carried out in an incubation buffer (50 mm sodium phosphate, pH 5.0) at 37 °C for 16 h. PVDF protein-binding membrane plates (Millipore) were used for removal of enzymes prior to HILIC-UPLC analysis.

### HILIC-UPLC

Fluorescently labeled glycans were separated by HILIC-UPLC using a 2.1 × 10-mm (1.7-μm particle size) ACQUITY® ethylene bridged hybrid glycan column (Waters) on a Waters ACQUITY® UPLC instrument. The following gradient was run: time = 0 min (*t* = 0): 22% A, 78% B (flow rate of 0.5 ml/min); *t* = 38.5: 44.1% A, 55.9% B (0.5 ml/min); *t* = 39.5: 100% A, 0% B (0.25 ml/min); *t* = 44.5: 100% A, 0% B; *t* = 46.5: 22% A, 78% B (0.5 ml/min), where solvent A was 50 mm ammonium formate, pH 4.4, and solvent B was acetonitrile. Fluorescence was measured using an excitation wavelength of 250 nm and a detection wavelength of 428 nm. A 2-AA–labeled glucose homopolymer ladder (Ludger) was used as a calibration standard for UPLC analysis of glycans. Data processing was performed using Empower 3 software. The percentage abundance of oligomannose- and complex-type glycans were calculated by integration of the relevant peak areas before and after endoglycosidase H digestion and following normalization.

### Receptor and complement binding assays

Methods describing the binding of mutants to tetrameric human DC-SIGN (Elicityl), C1q, and C5b-9 have been described previously (13, 15). The same ELISA protocol used to detect DC-SIGN binding was used for human Siglec-1, Siglec-4, and Siglec-3 (Sino Biologicals). ELISAs were used to investigate the binding of Fc mutants to human FcγRI, FcγRIIA, FcγRIIB, FcγRIIIA, and FcγRIIIB (Bio-Techne). Receptors were coated down to ELISA plates (Nunc) in carbonate buffer pH 9 (Sigma-Aldrich) at 2 μg/ml overnight at 4 °C, unless alternatively specified. The plates were blocked in PBS/0.1% Tween 20 (PBST) containing 5% dried skimmed milk. The plates were washed three times in PBST before adding Fc mutants at the indicated concentrations and left at 4 °C overnight. The plates were washed as above and incubated for 2 h with 1:500 dilution of an alkaline phosphatase-conjugated goat Fab′_2_ anti-human IgG (Jackson Laboratories). The plates were washed as above and developed for 15 min with 100 μl/well of a Sigmafast *p-*nitrophenyl phosphate solution (Sigma-Aldrich). The plates were read at 405 nm, and the data were plotted with GraphPad Prism.

## Author contributions

R. J. P. conceived and designed the overall study. R. J. P., P. A. B., N. L., J. A., and Y. W. designed and performed experiments. R. J. P. wrote the manuscript, and all authors commented on drafts and reviewed the final manuscript.

## Supplementary Material

Supplemental Data

## Footnotes

2 The abbreviations used are:

Fc: fragment crystallizable
IVIG: intravenous immunoglobulin
ITP: idiopathic thrombocytopenic purpura
tp: tail piece
HILIC: hydrophilic interaction chromatography
UPLC: ultra performance liquid chromatography
PNGase F: protein *N*-glycosidase F
2-AA: 2-aminobenzoic acid.
