# Supplementary material for: Engineering the fragment crystallizable (Fc) region of human IgG1 multimers and monomers to fine-tune interactions with sialic acid-dependent receptors
Source: J Biol Chem. 2017 Jun 15;292(31):12994–3007. doi: 10.1074/jbc.M117.795047 (PMC5546038; doi:10.1074/jbc.M117.795047)
Supplement: Supplemental Data [file supp_292_31_12994__index.html]

Engineering the fragment crystallizable (Fc) region of human IgG1 multimers and monomers to fine-tune interactions with sialic acid-dependent receptors — Engineering the Fc for sialic acid receptor interactions — Supplemental Data 

# Engineering the fragment crystallizable (Fc) region of human IgG1 multimers and monomers to fine-tune interactions with sialic acid-dependent receptors

## Supplemental Data

- Supplemental information (.pdf, 1.3 MB) - Supplemental Information
