# Supplementary material for: Engineering the fragment crystallizable (Fc) region of human IgG1 multimers and monomers to fine-tune interactions with sialic acid-dependent receptors
Source: J Biol Chem. 2017 Jun 15;292(31):12994–3007. doi: 10.1074/jbc.M117.795047 (PMC5546038; doi:10.1074/jbc.M117.795047)
Supplement: Supplemental Data [file 10.1074_M117.795047_jbc.M117.795047-1.pdf]

Supplementary information for:

Engineering the fragment crystallizable (Fc) region of human IgG1 multimers and monomers to fine-tune interactions with sialic acid dependent receptors

Patricia A Blundell[1], Ngoc Le[2], Joel Allen[2], Yasunori Watanabe[2], Richard J Pleass[1]

[1]Department of Parasitology, Liverpool School of Tropical Medicine, Pembroke Place, Liverpool, L3 5QA, UK. [2]Department of Biochemistry, University of Oxford, South Parks Road, Oxford, OX1 3QU, UK. Correspondence and requests for materials should be addressed to R.J.P. (email: richard.pleass@lstmed.ac.uk).

Supplementary Materials and Methods

Negative ion ESI MS/MS analysis of N-linked glycans

The identity of the glycans was confirmed by negative ion ESI MS/MS [1,2] using unlabeled glycans cleaned with a Nafion 117® membrane [3]. Static nano-electrospray mass spectrometry was performed with a Waters Synapt G2-Si HDMS ion mobility quadrupole-time-of-flight instrument. Samples in 1:1 (v/v) methanol:water containing 0.5 mM ammonium phosphate (to ensure maximum formation of phosphate adducts) were infused through platinum-palladium-coated borosilicate capillaries prepared in-house. The ion source conditions were: temperature, 80 °C; infusion needle potential, 1.2 kV; cone voltage 100 V. The traveling-wave ion-mobility cell (nitrogen) was operated with a wave velocity of 450 m s−1 and a wave height of 40 V. For MS/MS data acquisition, the parent ion was selected at low resolution (about 4 m/z mass window) and fragmented in the transfer cell with argon. The voltage on the collision cell was adjusted with mass to give an even distribution of fragment ions across the mass scale. Typical values were 80–120 V. Spectra (2 s scans) were acquired with a digitization rate of 4 GHz and accumulated until a satisfactory signal:noise ratio had been obtained. Other operating voltages were as recommended by the manufacturer. Instrument control, data acquisition, and processing were performed with MassLynx software Version 4.1, and Waters DriftScope software was used to extract singly charged glycan ions from the total profile and to reject MS/MS fragment ions that were not associated with the target glycan [3, 4]. Glycan fragments were labelled according to the scheme proposed by Domon and Costello [5].

[1] D.J. Harvey, L. Royle, C.M. Radcliffe, P.M. Rudd, R.A. Dwek, Structural and quantitative analysis of N-linked glycans by matrix-assisted laser desorption ionization and negative ion nanospray mass spectrometry, Analytical Biochemistry, 376 (2008) 44-60.
[2] E.V. Dixon, J.K. Claridge, D.J. Harvey, K. Baruah, X. Yu, S. Vasiljevic, S. Mattick, L.K. Pritchard, B. Krishna, C.N. Scanlan, J.R. Schnell, M.K. Higgins, N. Zitzmann, M. Crispin, Fragments of bacterial endoglycosidase S and immunoglobulin G reveal subdomains of each that contribute to deglycosylation, Journal of Biological Chemistry, 289 (2014) 13876-13889.
[3] K.O. Börnsen, M.D. Mohr, H.M. Widmer, Ion exchange and purification of carbohydrates on a Nafion® membrane as a new sample pretreatment for matrix-assisted laser desorption/ionization mass spectrometry, Rapid Communications in Mass Spectrometry, 9 (1995) 1031-1034.
[4] D.J. Harvey, C.A. Scarff, M. Edgeworth, M. Crispin, C.N. Scanlan, F. Sobott, S. Allman, K. Baruah, L. Pritchard, J.H. Scrivens, Travelling wave ion mobility and negative ion fragmentation for the structural determination of N-linked glycans, Electrophoresis, 34 (2013) 2368-2378.
[5] B. Domon, C.E. Costello, A systematic nomenclature for carbohydrate fragmentations in FAB-MS/MS spectra of glycoconjugates, Glycoconj J, 5 (1988) 397-409.

A)

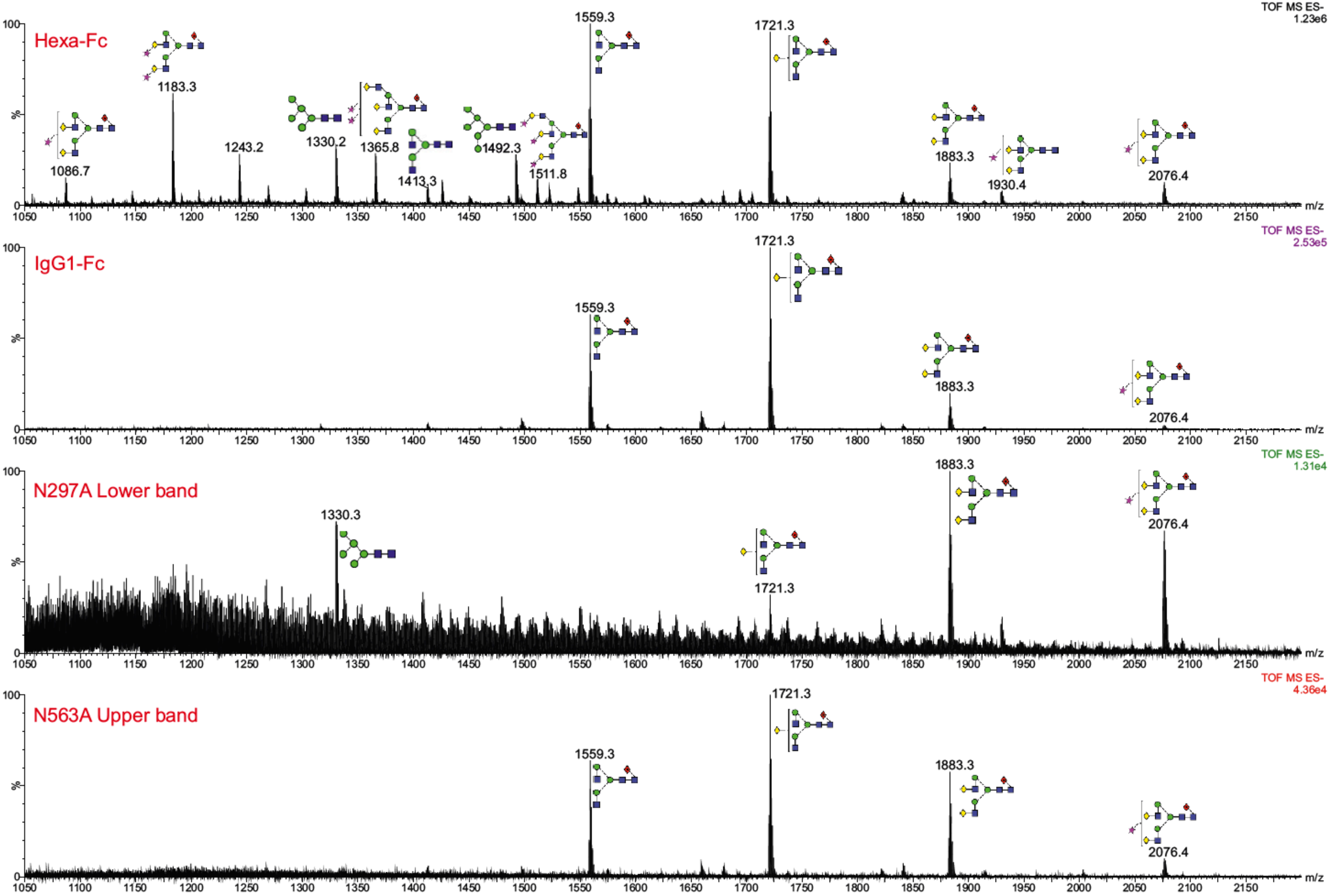

Hexa-Fc
TOF MS ES-
1.23e6
1086.7
1183.3
1243.2
1330.2
1365.8
1413.3
1492.3
1511.8
1559.3
1721.3
1883.3
1930.4
2076.4
IgG1-Fc
TOF MS ES-
2.53e5
1559.3
1721.3
1883.3
2076.4
N297A Lower band
TOF MS ES-
1.31e4
1330.3
1721.3
1883.3
2076.4
N563A Upper band
TOF MS ES-
4.36e4
1559.3
1721.3
1883.3
2076.4
100
%
0
m/z
1050
1100
1150
1200
1250
1300
1350
1400
1450
1500
1550
1600
1650
1700
1750
1800
1850
1900
1950
2000
2050
2100
2150

B)

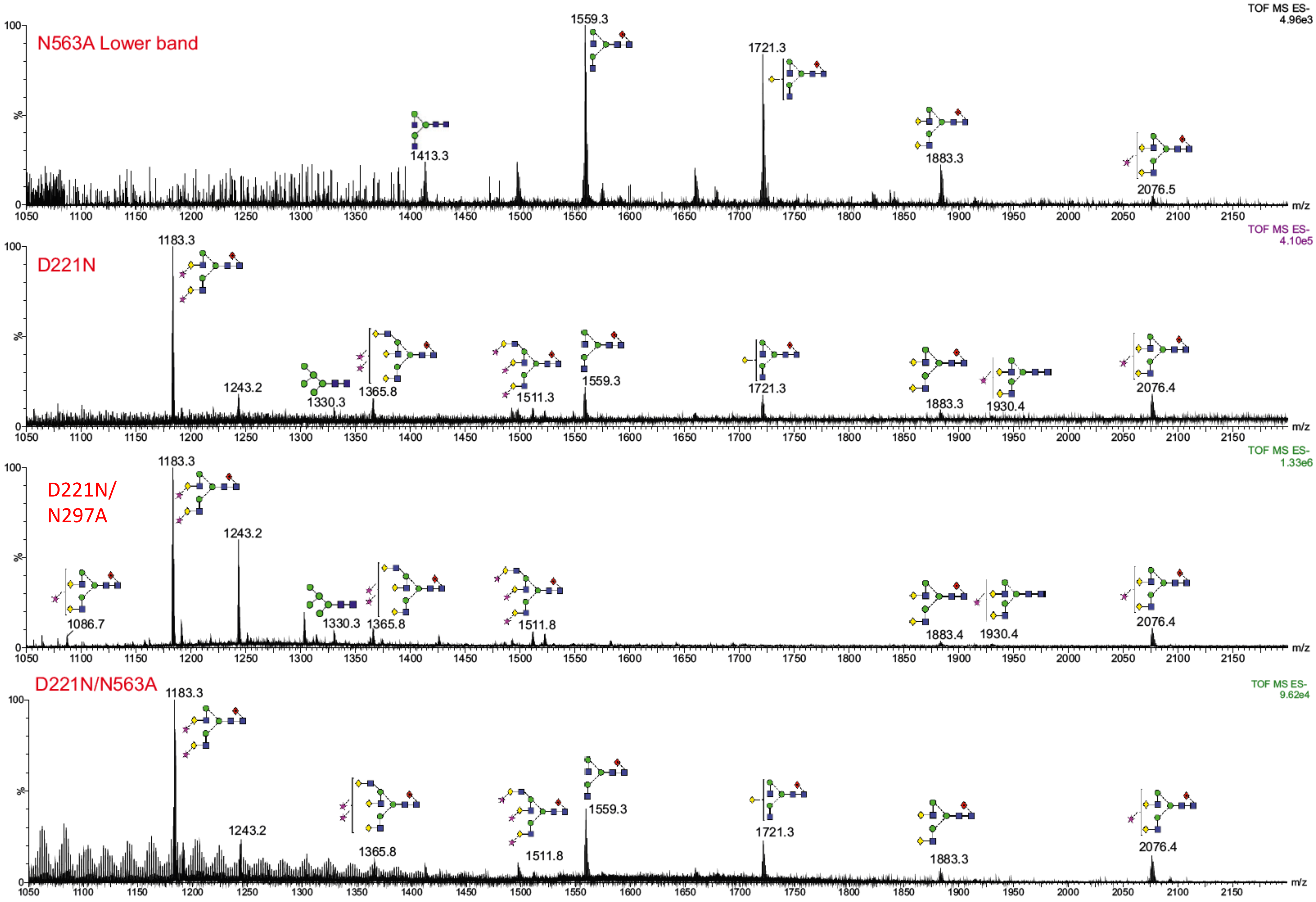

N563A Lower band
TOF MS ES-
4.96e3
1559.3
1721.3
1413.3
1883.3
2076.5
D221N
TOF MS ES-
4.10e5
1183.3
1243.2
1330.3
1365.8
1511.3
1559.3
1721.3
1883.3
1930.4
2076.4
D221N/
N297A
TOF MS ES-
1.33e6
1183.3
1243.2
1086.7
1330.3
1365.8
1511.8
1883.4
1930.4
2076.4
D221N/N563A
TOF MS ES-
9.62e4
1183.3
1243.2
1365.8
1511.8
1559.3
1721.3
1883.3
2076.4
100
%
0
m/z

C)

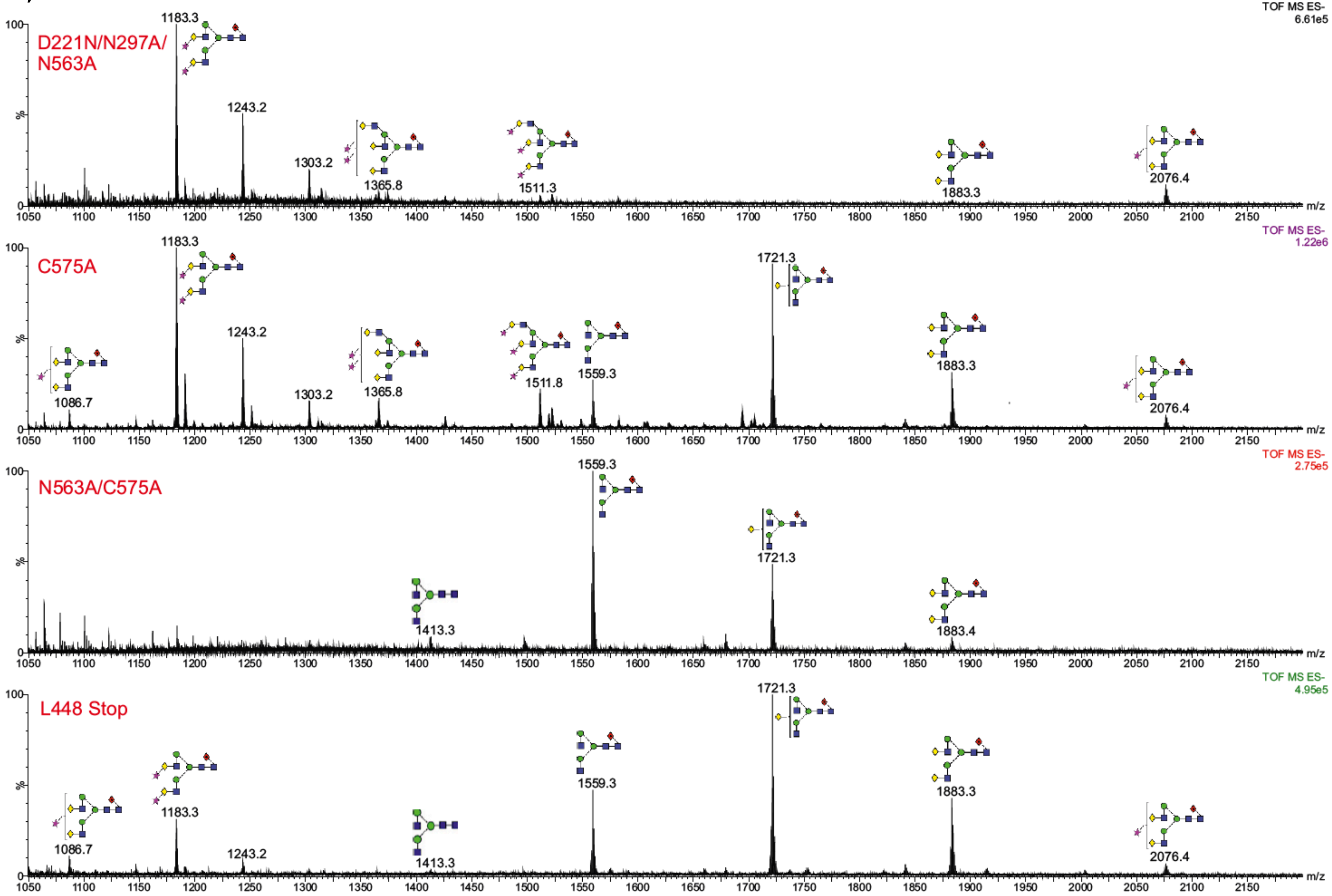

D221N/N297A/
N563A
TOF MS ES-
6.61e5
1183.3
1243.2
1303.2
1365.8
1511.3
1883.3
2076.4
C575A
TOF MS ES-
1.22e6
1086.7
1183.3
1243.2
1303.2
1365.8
1511.8
1559.3
1721.3
1883.3
2076.4
N563A/C575A
TOF MS ES-
2.75e5
1413.3
1559.3
1721.3
1883.4
L448 Stop
TOF MS ES-
4.95e5
1086.7
1183.3
1243.2
1413.3
1559.3
1721.3
1883.3
2076.4
100
%
0
m/z
1050
1100
1150
1200
1250
1300
1350
1400
1450
1500
1550
1600
1650
1700
1750
1800
1850
1900
1950
2000
2050
2100
2150

D)

Samples indicating that glycans are absent

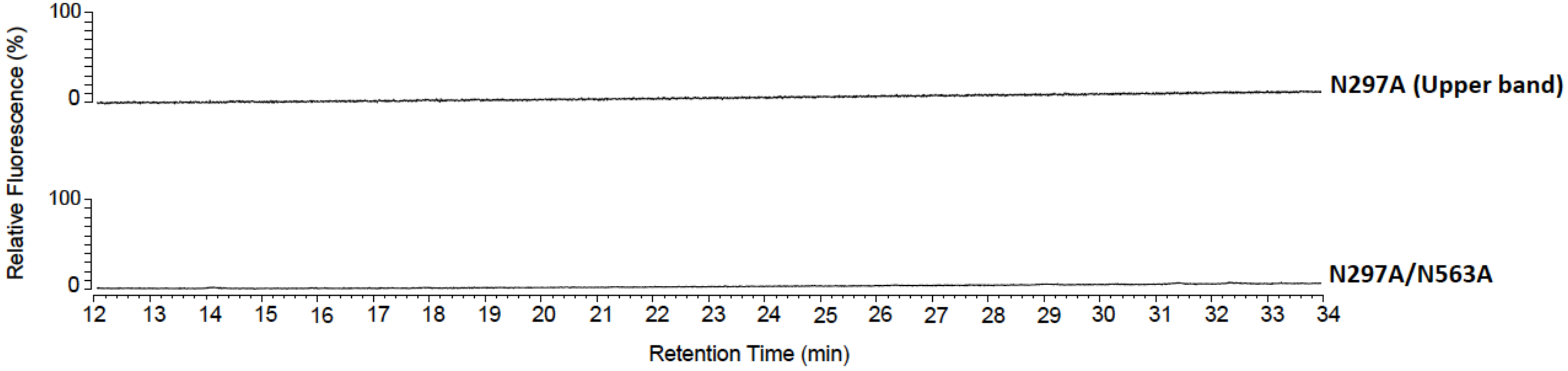

Samples with low signal - undetermined

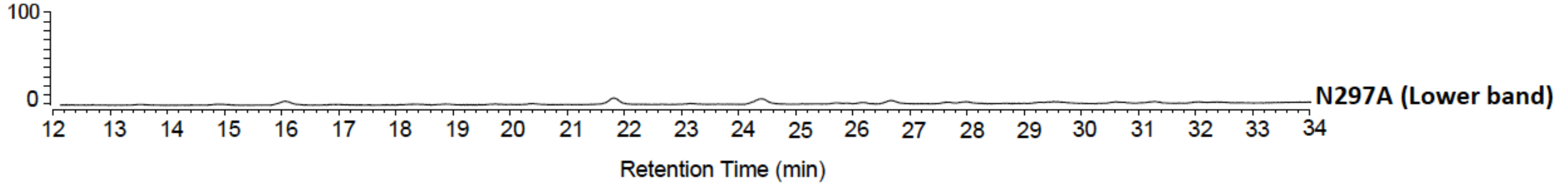

Figure S1. MALDI-TOF mass spectrometric analysis of N-linked glycans released from hexa-Fc variants. Panel A) above for hexa-Fc, IgG1-Fc, N297A (lower band), N563A (upper band); panel B) above for N563A (lower band), D221N, D221N/N297A, and D221N/N563A; panel C) above for D221N/N297A/N563A, C575A, N563A/C575A and L448STOP; panel D) above for N297A and N297A/N563A. N-linked glycans were released from IgG-Fc by PNGase F digestion. The symbolic representation of glycans follows that of Harvey et al [refs 1,4] with residues in both the schematic diagrams and molecular graphics following the Colour scheme of the Consortium for Functional Glycomics. Masses, compositions and structures of the N-glycans are shown in Table S1 below.

Table S1. List of masses, compositions and structures of the N-glycans derived from electrospray mass spectrometry and then converted into singly and doubly charged ions using ion mobility extraction.

List of N-glycan structures detected by ESI-MS

m/z	Ion[1]	Composition				Structure[2]	Samples[3]										
		Hex	HexNAc	Fuc	Neu5Ac		Hexa-Fc	IgG-Fc	N563A (Upper band)	N563A (Lower band)	D221N	D221N/ N297A	D221N/ N563A	D221N/ N297A/ N563A	C575A	N563A/ C575A	L448Stop
1330.2	a	5	2	0	0	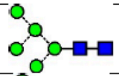	Y	-	-	-	Y	Y	-	-	-	-	-
1413.3	a	3	4	0	0	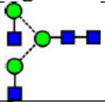	Y	-	-	Y	Y	-	Y	-	-	Y	Y
1493.5	a	6	2	0	0	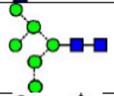	Y	-	-	-	Y	-	-	-	-	-	-
1559.3	a	3	4	1	0	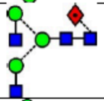	Y	Y	Y	Y	Y	-	Y	-	Y	Y	Y
1721.3	a	4	4	1	0	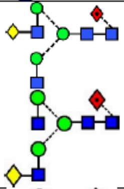	Y	Y	Y	Y	Y	-	Y	-	Y	Y	Y
1883.3	a	5	4	1	0	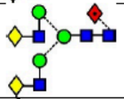	Y	Y	Y	Y	Y	Y	Y	Y	Y	Y	Y
1086.7	c				1	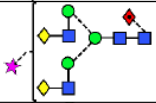	Y	Y	Y	Y	Y	Y	Y	Y	Y	-	Y
2076.4	b																
1930.4	b	5	4	0	1	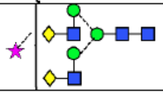	Y	-	-	-	Y	Y	-	-	-	-	-

1183.3	d	5	4	1	2	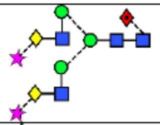	Y	-	-	-	Y	Y	Y	Y	Y	-	Y
1365.8	d	6	5	1	2	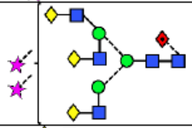	Y	-	-	-	Y	Y	Y	Y	Y	-	-
1511.8	d	6	5	1	3	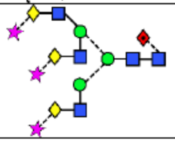	Y	-	-	-	Y	Y	Y	Y	Y	-	-

1. Ions:

a = $[M+H_2PO_4]^-$

b = $[M-H]^-$

c = $[M-H+(H_2PO_4)]^{2-}$

d = $[M-H_2]^{2-}$

2. Symbols for monosaccharide residues: ■ = GlcNAc, ● = mannose, ◆ = galactose, ◆ = fucose, ★ = Neu5Ac (sialic acid).

3. 'Y' indicates that the structure was present in the sample.

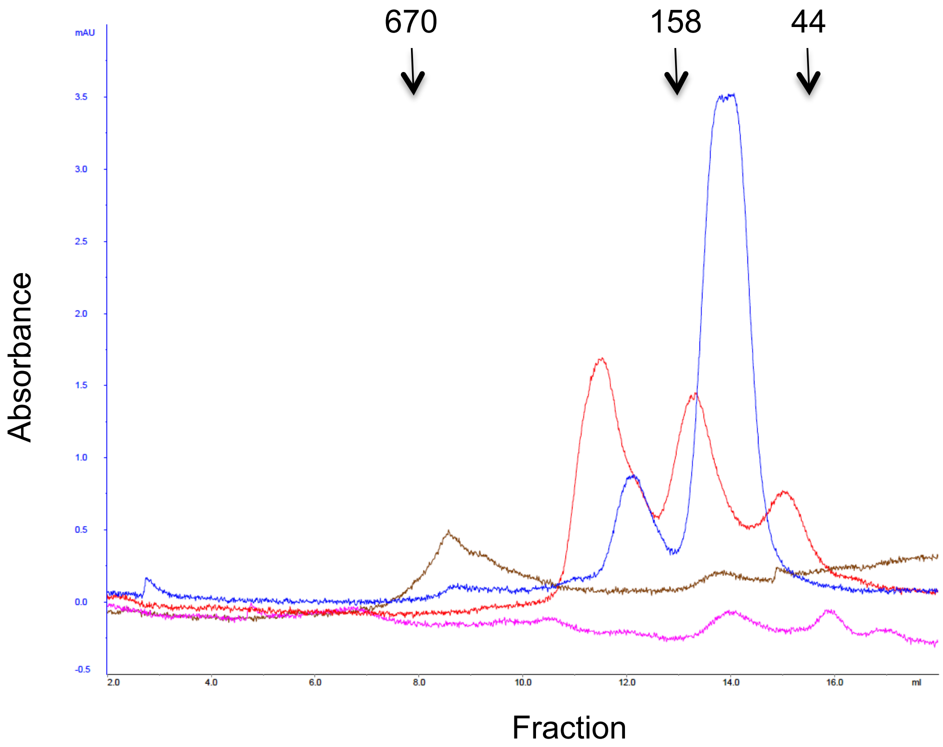

Fig. S2. Size-exclusion chromatography (SEC) analysis. Samples run on Superdex-200 10/300GL column for hexa-Fc (red trace), and N563A (brown trace), C575A (blue trace), N563A/C575A (pink trace) mutants. Elution profiles of molecular weight standards are omitted for clarity but indicated with arrows for bovine thyroglobulin (670 kDa), bovine γ-globulin (158 kDa) and chicken ovalbumin (44 kDa).

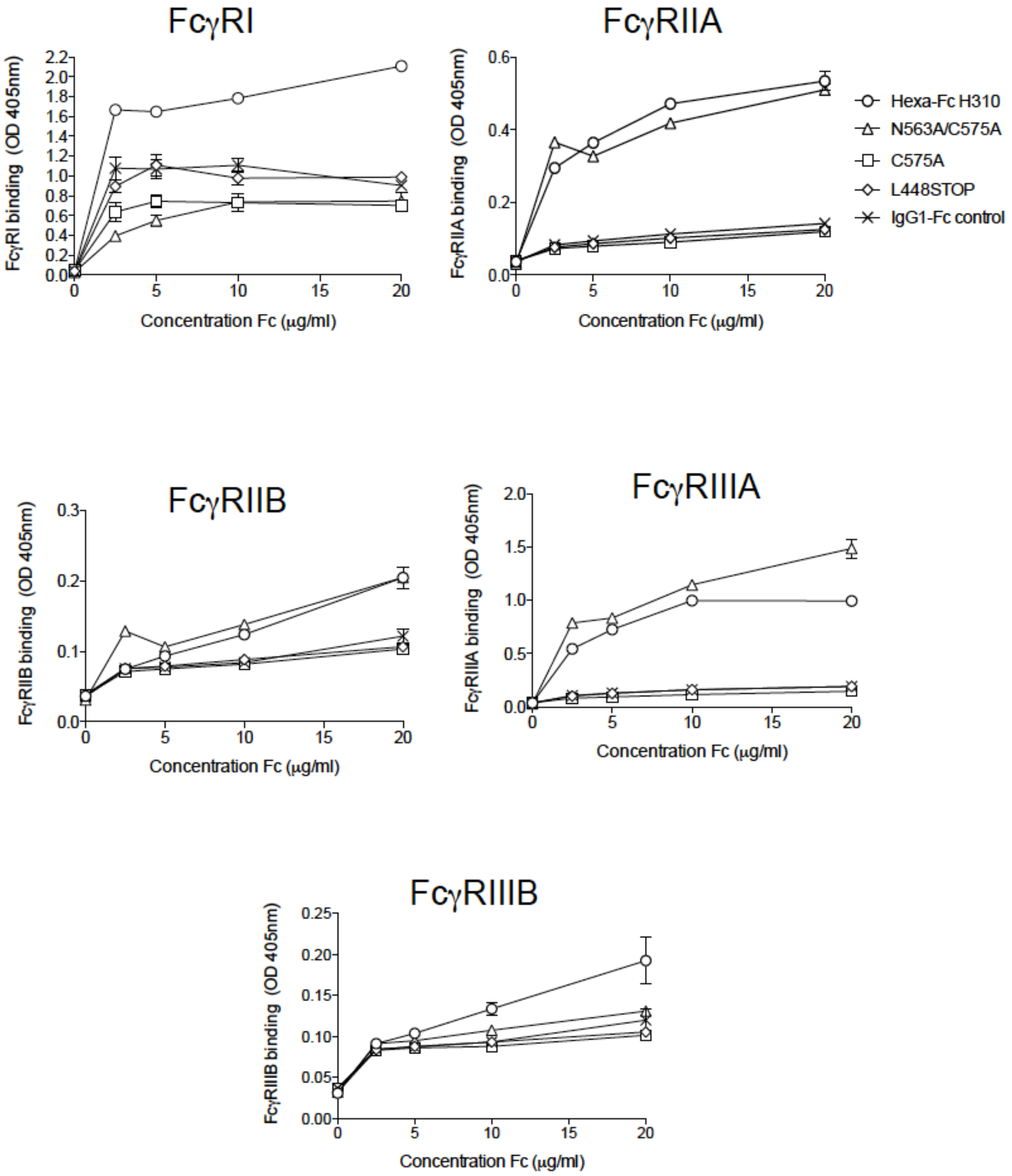

Fig. S3. Binding of selected N-linked glycan variants including C575A monomer to classical FcγRs assessed by ELISA. Data shown from one of two independent experiments.

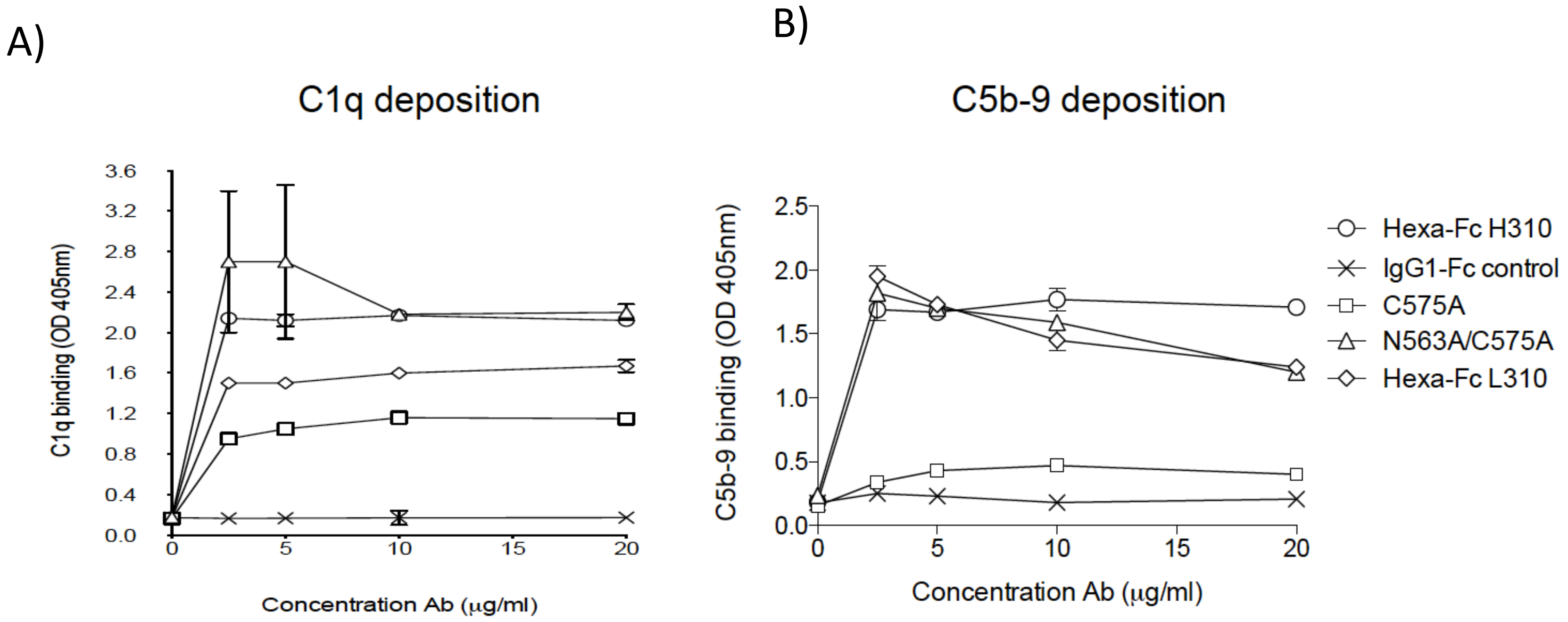

Fig. S4. Binding of variants to complement assessed by ELISA. (A) C1q binding and (B) C5b-9 deposition. Although the C575A monomer can still bind C1q complement activation as judged by C5b-9 deposition is poor. Data shown from one of two independent experiments.

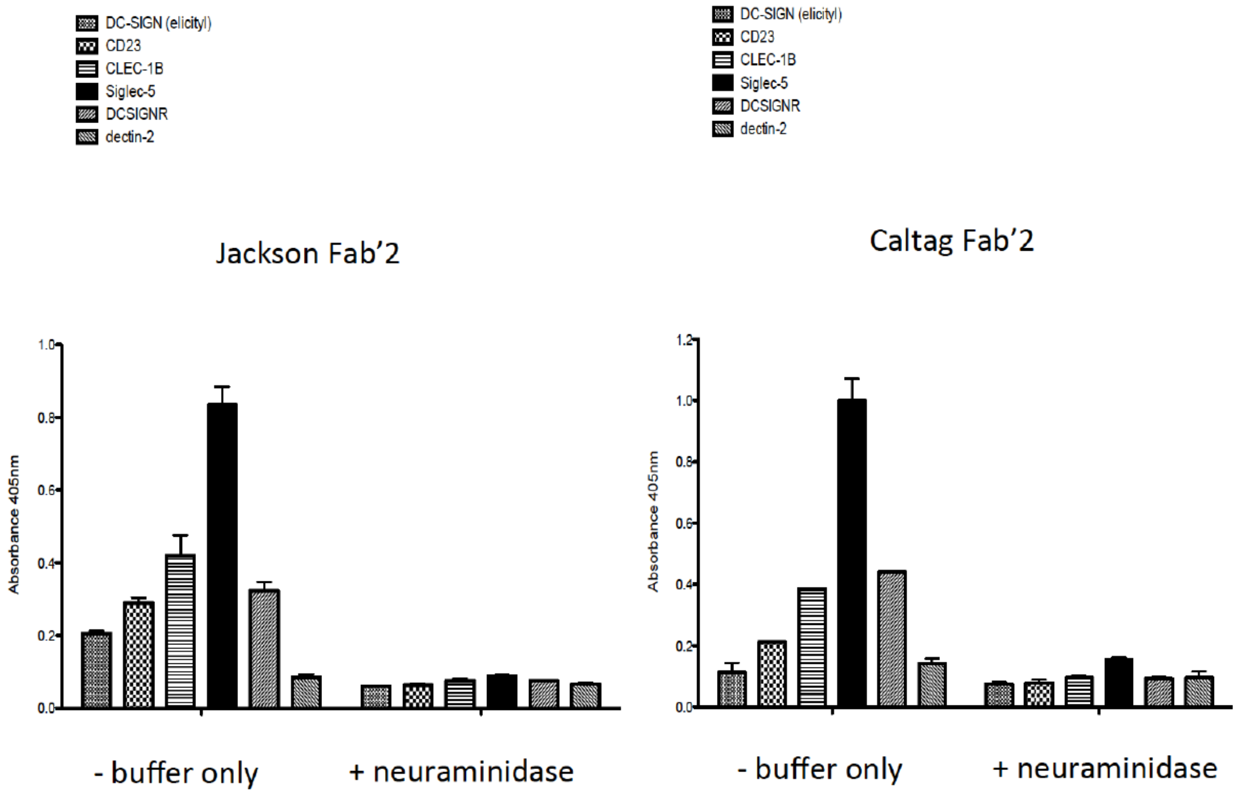

Fig. S5. Goat Fab'2 binds directly to Siglec-5 through a glycan dependent mechanism. Glycan receptors from Thermo Fisher (Sino Biological) were coated down at 5 μg/ml in carbonate buffer prior to blocking in TMS / 5% BSA as described in methods. Five micrograms of Fab'$_2$ from two different manufacturers was treated with or without α2-3,6,8 neuraminidase in a 20 μl final volume (New England Biological) as per manufacturers instructions. After digesting at 37°C for 4h the mix was made up to 1.2 ml with TMS buffer prior to adding to coated down receptors. ELISAs were then treated and developed with substrate as described in the methods.
